# Supplementary material for: Diurnal pattern of respiration in corals and algae and its implications for gross primary production quantification
Source: PLoS One. 2025 Jun 27;20(6):e0326146. doi: 10.1371/journal.pone.0326146 (PMC12204508; doi:10.1371/journal.pone.0326146)
Supplement: S4 Data — (PDF) [file pone.0326146.s004.pdf]

Diurnal pattern of respiration by corals and algae and its implications for gross primary production quantification

Authors: Yvonne Sawall, Roderick Bakker, Natalia E. Padillo-Anthemides, Nicole Adamson

Supplementary Information

METHODS:

Generalized additive model – respiration rates (R) at sunrise and sunset

Table S1: GAM-derived respiration rates (R; [µmol O_2 cm^{-2} h^{-1}]) at sunrise and sunset. These values were added to the raw data sets of the net photosynthesis rates (NP) and attributed with a weighing factor of 100 to force the GAMs of NP through these points.

	Sunrise	Sunset	Sunrise	Sunset	Sunrise	Sunset
	Caulerpa		***Ceramium***		***Laurencia***	
Fall	-1.41	-1.627	-1.265	- 1.643	-1.01	-1.467
	Diploria		***Porites***		***Montastrea***	
Fall	-0.67	-0.90	-0.587	-0.79	-0.512	-0.75
Spring	-1.08	-1.505	-1.20	-1.52	-1.06	-1.59
Summer	-1.75	-1.646	-1.70	-1.806	-1.53	-2.007

Diurnal pattern of respiration by corals and algae and its implications for gross primary production quantification

Authors: Yvonne Sawall, Roderick Bakker, Natalia E. Padillo-Anthemides, Nicole Adamson

Algal species and surface area determination

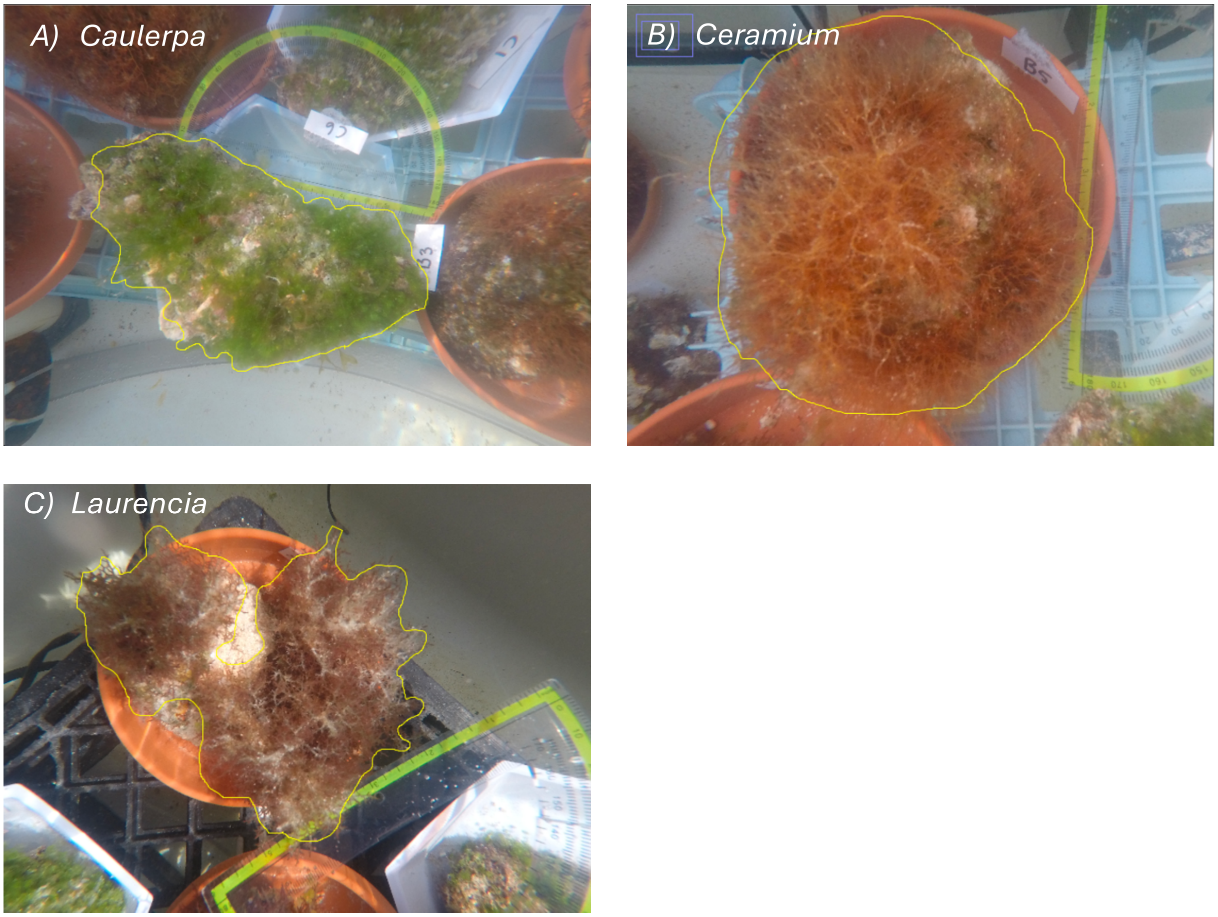

Fig. S1: Examples of individual algae assemblages dominated by *Caulerpa* (A), *Ceramium* (B) and *Laurencia* (C). The yellow lines are drawn manually in the software ImageJ to determine the 2D surface are of the respective algae assemblage. The ruler in the pictures is for size reference.

Diurnal pattern of respiration by corals and algae and its implications for gross primary production quantification

Authors: Yvonne Sawall, Roderick Bakker, Natalia E. Padillo-Anthemides, Nicole Adamson

<u>Oxygen sensor comparisons - FireSting versus MiniDOT:</u>

Two FireSting and two MiniDOT oxygen sensors were placed simultaneously in 2 incubation chambers containing corals that photosynthesized. A total of 5 corals were incubated in consecutive order (coral A-E). Figure S2 shows the raw data of the oxygen concentrations measured over a 30-minute period in the 2 chambers, for 2 out of 5 corals. While the FireSting registered higher O_2 concentrations than the MiniDOT, due to different calibrations, the slopes are very similar. Also, the 2-point measurements (start and end; FireSting) versus continues measurements (every minute; MiniDOT) did not show a difference in the slope. Figure S3 shows the data of all 5 corals combined. A paired two-way T-test with a Bonferroni correction to account for repeated sampling of the corals in multiple incubations (n = 5) did not show a difference of the slopes between the 2 sensors ($p > 0.05$). Based on these results it was concluded that the two sensors can be used interchangeably for our experiment.

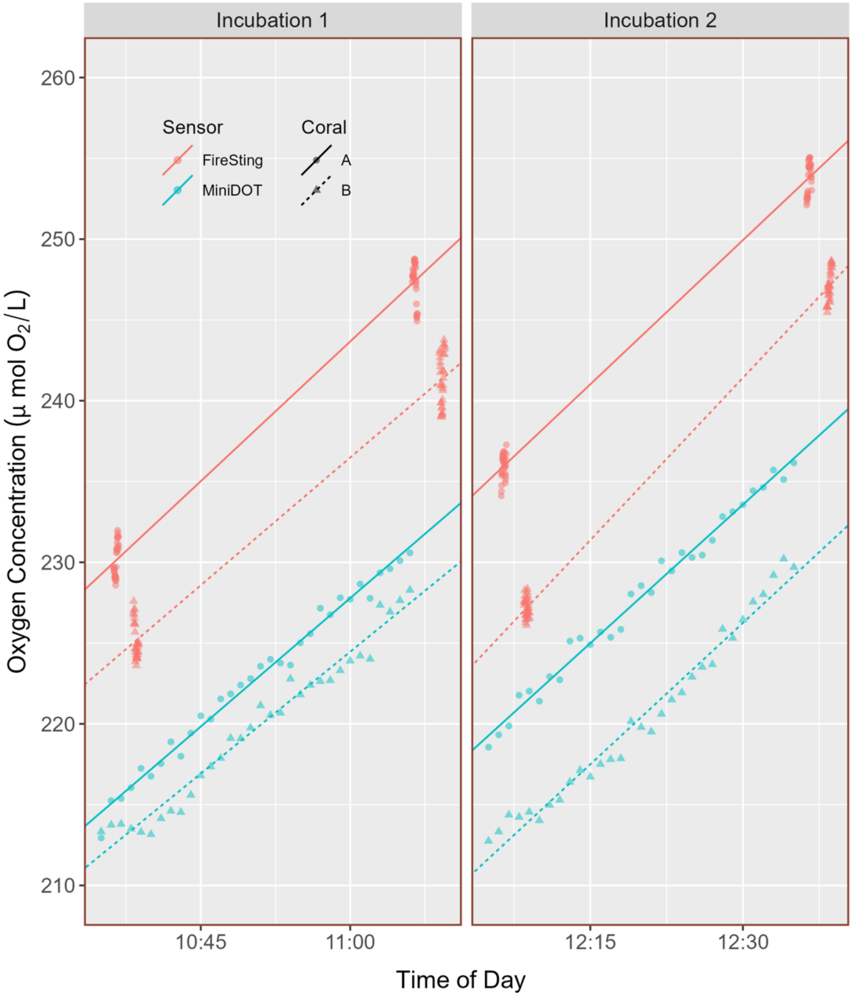

Figure S2: Oxygen concentration in the incubation chambers measured by the FireString (red) and the MiniDOT (blue) simultaneously. FireSting measurements were taken every second for 30 sec at the start and end of incubations. MiniDOT measurements were taken every minute throughout the incubation. T out for 5 corals are shown as examples.

Diurnal pattern of respiration by corals and algae and its implications for gross primary production quantification

Authors: Yvonne Sawall, Roderick Bakker, Natalia E. Padillo-Anthemides, Nicole Adamson

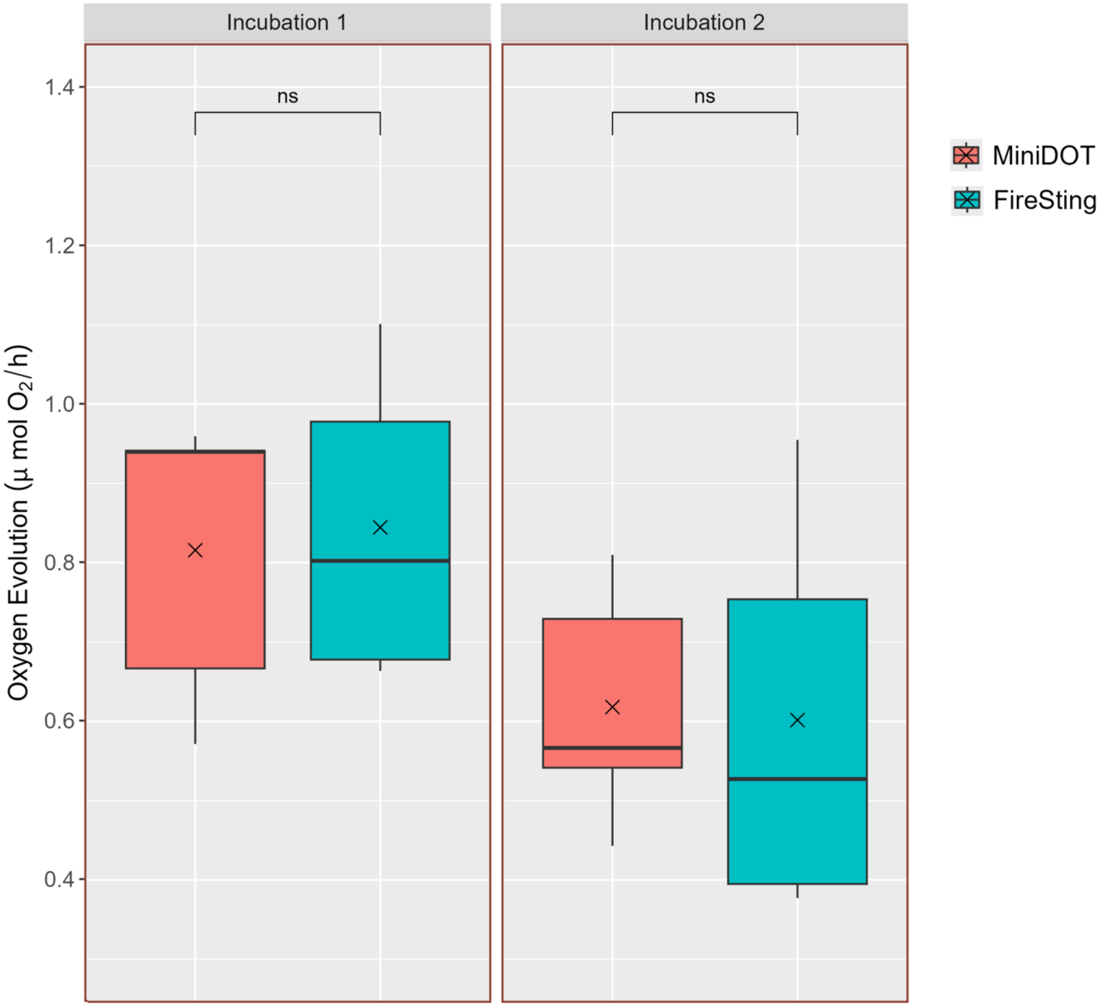

Figure S3: Oxygen evolution rates measured with the FireSting and MiniDOT. Horizontal bars in boxplots indicate median values and black "x" indicate mean values. ns = non-significant difference ($p>0.05$) between the oxygen evolution rates derived from the FireSting versus the MiniDOT. n=5 corals.

Diurnal pattern of respiration by corals and algae and its implications for gross primary production quantification

Authors: Yvonne Sawall, Roderick Bakker, Natalia E. Padillo-Anthemides, Nicole Adamson

RESULTS

Table S2: Generalized additive model (GAM) summary statistics of respiration (top) and net photosynthesis (bottom). Family: Gaussian. Dev. explained = Deviance explained. EDF = effective degrees of freedom (represents the complexity of the smooth). F value is an approximate value that describes, in simple terms, the differences between the smooth and a horizontal line through the 95% confidence interval. The p-value indicates if the difference between the smooth and a horizontal line is significantly different.

**Respiration**					
**Fall**	**R squared**	**Dev. explained**	**EDF**	**F value**	**p value**
Algae					
Caulerpa	0.183	0.206	6.54	6.92	0.000
Ceramium	0.100	0.120	6.60	4.28	8.33E-05
Laurencia	0.256	0.271	6.72	14.81	0.000
Coral					
Diploria	0.228	0.242	7.11	14.54	0.000
Porites	0.210	0.226	7.07	11.89	0.000
Montastrea	0.290	0.304	7.86	19.06	0.000
**Spring**					
Diploria	0.152	0.172	4.84	6.33	6.48E-06
Porites	0.286	0.313	5.89	8.87	0.000
Montastrea	0.232	0.254	5.33	9.03	0.000
**Summer**					
Diploria	0.045	0.067	2.65	1.59	0.185
Porites	0.288	0.339	4.46	4.84	0.001
Montastrea	0.238	0.274	5.04	5.51	5.20E-05

**Net photosynthesis**					
**Fall**	**R squared**	**Dev. explained**	**EDF**	**F value**	**p value**
Algae					
Caulerpa	0.577	0.625	5.03	75.52	0.000
Ceramium	0.731	0.767	6.23	136.26	0.000
Laurencia	0.609	0.677	5.51	93.44	0.000
Coral					
Diploria	0.635	0.678	5.43	108.86	0.000
Porites	0.637	0.682	6.03	85.61	0.000
Montastrea	0.676	0.717	5.72	121.44	0.000
Spring					
Diploria	0.559	0.569	5.38	48.22	0.000
Porites	0.415	0.425	4.55	33.83	0.000
Montastrea	0.492	0.502	4.95	39.44	0.000
Summer					
Diploria	0.623	0.699	3.84	110.38	0.000
Porites	0.486	0.602	3.57	51.76	0.000
Montastrea	0.564	0.656	3.64	74.13	0.000

Diurnal pattern of respiration by corals and algae and its implications for gross primary production quantification

Authors: Yvonne Sawall, Roderick Bakker, Natalia E. Padillo-Anthemides, Nicole Adamson

Table S3: Summary of metabolic rates. *R* = respiration rate. new = considers day-night variability. trad = traditional, based on nighttime *R* only. *GP* = gross photosynthesis rate. *NP* = net photosynthesis rate. All rates are derived from the fitted Generalized Additive Models (GAMs) and are presented as mean (confidence interval).

			Hourly rates		Daily rates						
			24-h period				Sunrise to sunset				
			R minimum	*R* maximum	***R* new**	*R* trad	***GP* new**	*GP* trad	*NP*	***R* new(day)**	*R* trad(day)
Taxa	Genus	Season	[µmol O_2 cm^{-2} h^{-1}]		[µmol O_2 cm^{-2} d^{-1}]					[µmol O_2 cm^{-2} $daytime^{-1}$]	
Algae	*Caulerpa*	Fall	1.35 (0.17)	2.08 (0.21)	38.9 (4.1)	34.2 (3.6)	50.1 (9.6)	45.4 (8.7)	28.6 (7.6)	21.4 (2.0)	16.7 (1.8)
Algae	*Ceramium*	Fall	1.27 (0.13)	1.92 (0.20)	37.4 (3.8)	35.4 (3.6)	38.4 (6.4)	36.3 (6.0)	19.0 (4.4)	19.4 (2.0)	17.3 (1.8)
Algae	*Laurencia*	Fall	0.81 (0.19)	1.94 (0.19)	30.7 (3.7)	23.5 (2.8)	41.0 (6.6)	33.8 (5.4)	22.3 (4.7)	18.7 (1.8)	11.5 (1.4)
Coral	*Diploria*	Fall	0.60 (0.11)	1.22 (0.10)	19.9 (2.1)	16.0 (1.7)	24.2 (4.0)	20.2 (3.3)	12.4 (3.0)	11.8 (1.0)	7.8 (0.8)
Coral	*Montastrea*	Fall	0.50 (0.70)	1.10 (0.08)	17.0 (1.7)	13.8 (1.4)	20.1 (3.3)	17.0 (2.7)	10.2 (2.4)	9.9 (0.9)	6.8 (0.7)
Coral	*Porites*	Fall	0.55 (0.10)	1.08 (0.10)	17.7 (1.9)	14.3 (1.6)	22.9 (4.4)	19.6 (3.8)	12.6 (3.5)	10.4 (1.0)	7.0 (0.8)
Coral	*Diploria*	Spring	1.05 (0.17)	1.62 (0.11)	32.1 (3.6)	29.1 (3.0)	33.3 (5.5)	30.4 (5.0)	14.5 (4.1)	18.8 (1.5)	15.8 (1.7)
Coral	*Montastrea*	Spring	1.02 (0.19)	1.83 (0.14)	33.7 (4.0)	29.3 (3.4)	36.0 (6.6)	31.5 (5.8)	15.6 (4.8)	20.4 (1.8)	15.9 (1.9)
Coral	*Porites*	Spring	1.17 (0.20)	1.89 (0.12)	34.7 (3.8)	30.3 (3.3)	35.5 (6.2)	31.0 (5.5)	14.6 (4.6)	20.9 (1.6)	16.6 (1.8)
Coral	*Diploria*	Summer	1.58 (0.17)	1.82 (0.12)	40.5 (3.1)	40.1 (3.1)	50.2 (8.5)	48.9 (8.3)	25.9 (6.8)	24.4 (1.7)	23.0 (1.8)
Coral	*Montastrea*	Summer	1.44 (0.40)	2.86 (0.39)	49.2 (8.7)	39.1 (6.9)	60.3 (13.7)	49.3 (11.2)	26.8 (8.9)	33.5 (4.8)	22.5 (4.0)
Coral	*Porites*	Summer	1.39 (0.39)	2.44 (0.33)	43.8 (7.4)	36.3 (6.1)	67.9 (17.0)	59.5 (14.9)	38.7 (12.8)	29.2 (4.1)	20.8 (3.5)

Diurnal pattern of respiration by corals and algae and its implications for gross primary production quantification

Authors: Yvonne Sawall, Roderick Bakker, Natalia E. Padillo-Anthemides, Nicole Adamson

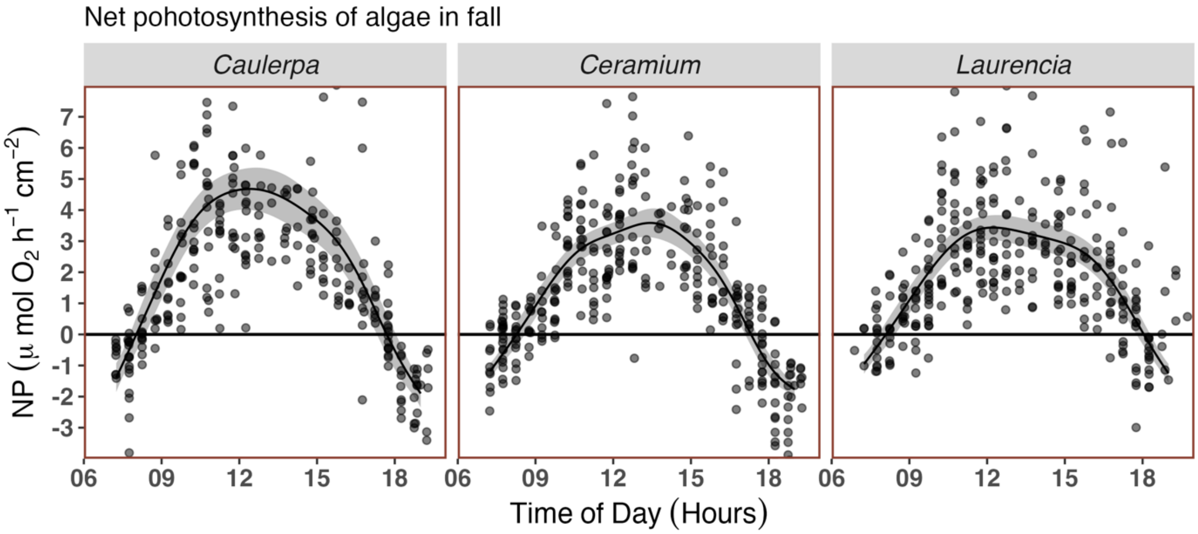

Fig. S4: Hourly net photosynthesis rates of all experimental algae specimens in fall. Different color intensity of dots represents different replicates (n=9). Curve fitting: Generalized additive model (GAM) and 95% confidence interval.

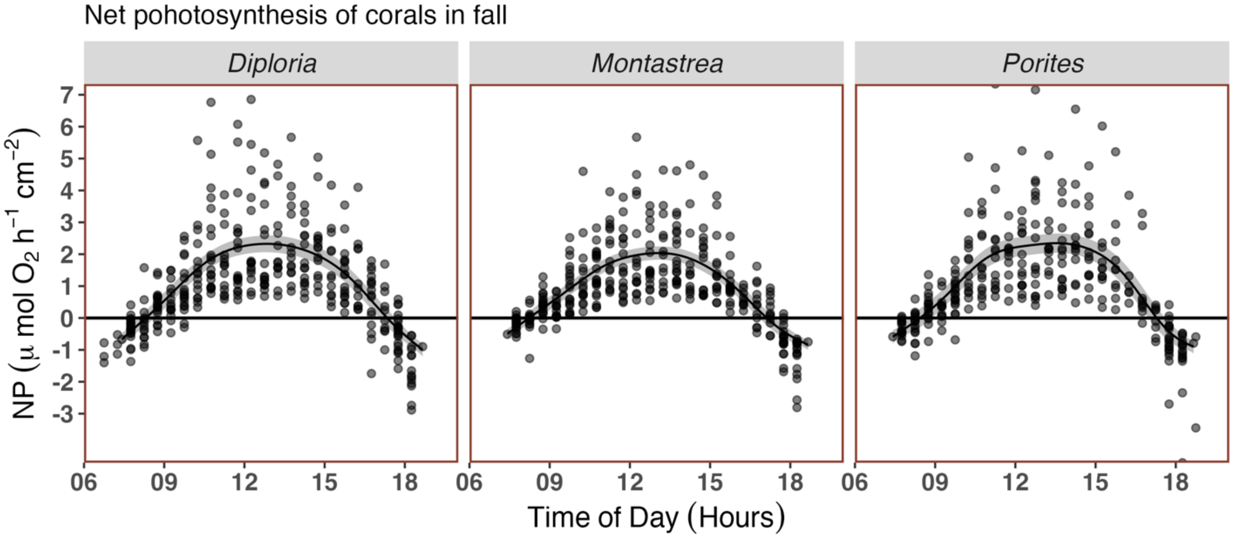

Fig. S5: Hourly net photosynthesis rates of all experimental corals in fall. Different color intensity of dots represents different replicates (n=9). Curve fitting: Generalized additive model (GAM) and 95% confidence interval.

Diurnal pattern of respiration by corals and algae and its implications for gross primary production quantification

Authors: Yvonne Sawall, Roderick Bakker, Natalia E. Padillo-Anthemides, Nicole Adamson

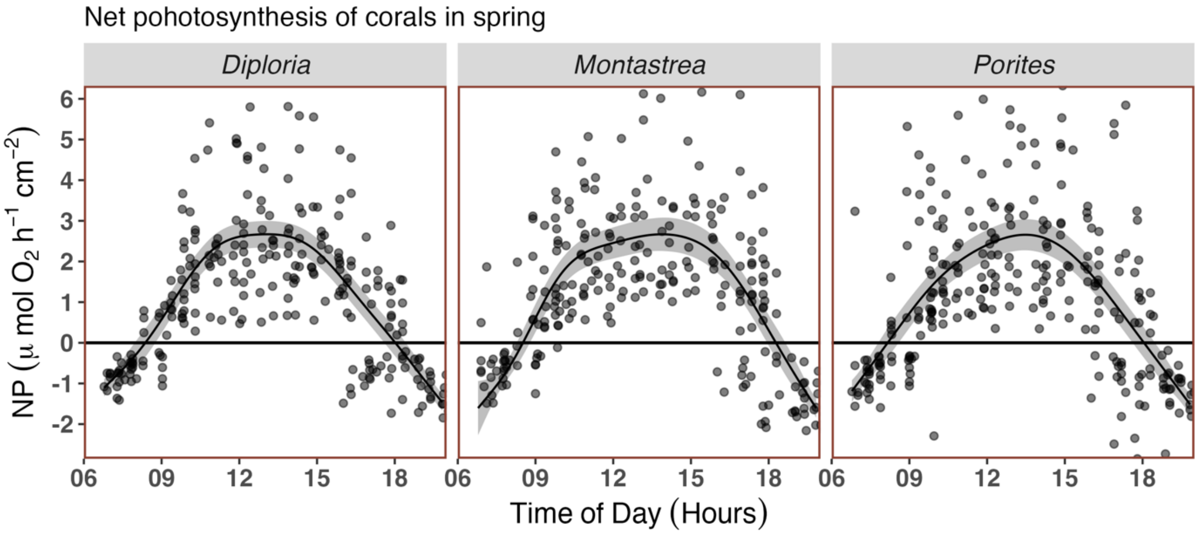

Fig. S6: Hourly net photosynthesis rates of all experimental corals in spring. Different color intensity of dots represents different replicates (n=7-9). Curve fitting: Generalized additive model (GAM) and 95% confidence interval.

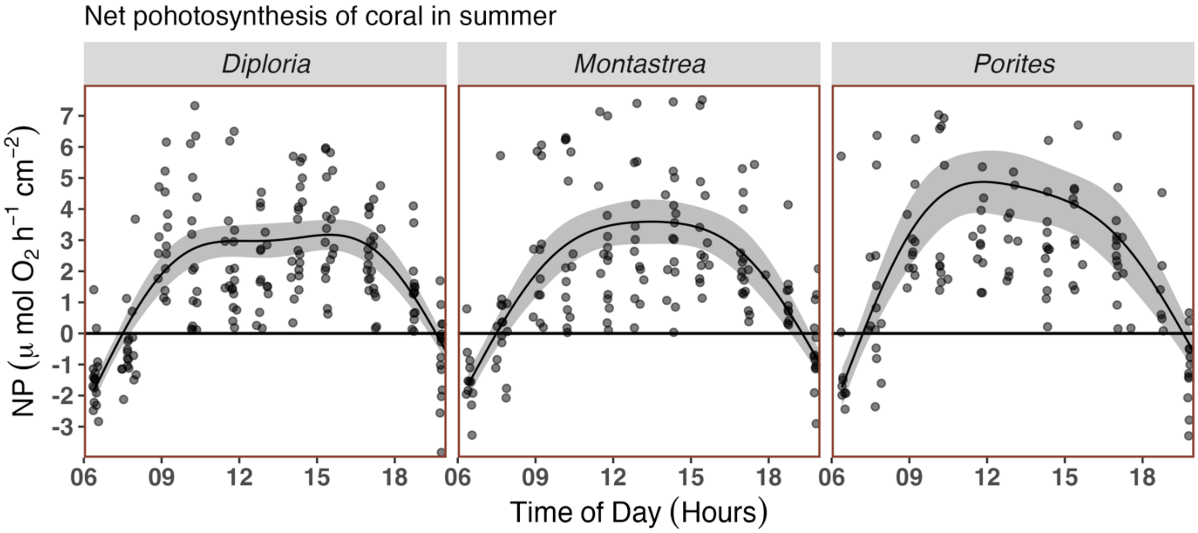

Fig. S7: Hourly net photosynthesis rates of all experimental corals in summer. Different color intensity of dots represents different replicates (n=7-9). Curve fitting: Generalized additive model (GAM) and 95% confidence interval.
