# Supplementary material for: Diurnal pattern of respiration in corals and algae and its implications for gross primary production quantification
Source: PLoS One. 2025 Jun 27;20(6):e0326146. doi: 10.1371/journal.pone.0326146 (PMC12204508; doi:10.1371/journal.pone.0326146)
Supplement: Attachment — Submitted filename: Rebuttal Letter PONE.docx [file pone.0326146.s005.docx]

**Rebuttal Letter PONE-D-24-55887**

First of all, we want to that the editor and reviewers for their constructive criticism. We have taken into consideration almost all critics / suggestion and we believe the manuscript has improved substantially.

**The reviewers' main comments and suggestions include:**

-    Additional analyses appear for specific subsets of data (comparison of measured oxygen fluxes between new and traditional methods)

**RE**: We are not able to conduct statistical analyses for the comparisons between results of the new and traditional approach. As outlined in our response reviewer #1 (L318-339), the daily rates are a based on the GAMs that were fitted across all (!) replicates. As described in the methods section “2.6 curve fitting and error propagation” it was not possible to do GAMs on individual coral or algae specimen as there were not enough data points per specimen/replicate. Consequently, we don’t have derived values, such as GP, GP/R ratios, maximum R, etc. from individuals which precludes statistical analyses.

We tested, however, if the GAM models are significantly different to a straight horizontal line with the p-values being presented in table S1, and now also reported in the revised ms. All diurnal patters of R were significantly different from a straight horizontal line except for *Diploria* in summer. All diurnal patterns of NP were significantly different from a straight horizontal line.

-    Additional supplementary data to support certain statements in the manuscript

**RE**: We added the results of the comparisons between oxygen flux measurements to the supplementary information (Fig. S2 and S3). We also provided pictures of algae specimen and how we determined surface area (Fig. S1).

-    A schematic representation of the methodology (for details, please refer to detailed comments below)

**RE**: We added a new figure that (hopefully) clarifies the meaning of the different rates calculated and their acronyms (Fig. 1).

-    More detailed discussion and research recommendations based on Rnew - specifically: what are the implications of potentially higher turnover rates in algae and corals (e.g., for energy budgets)

**RE**: We expanded the discussion to address this in more detail. The paragraph explaining this specifically, now reads:

L531-542: “Those turnover rates reflect the scale of metabolic activity, including, for example, energy requirements of transmembrane transporters, movement, and cellular maintenance processes such as protein turnover and repair mechanisms. Quantifying these rates is important because their demand can vary and adjust in response to changing environmental conditions. Species or genotypes with high energy turnover—and/or a greater capacity to regulate their energy turnover in response to changing demands—may be better equipped to cope with environmental stressors such as ocean warming and acidification. For instance, some corals can increase the activity of energy-demanding H⁺-pumps at the site of calcification to maintain elevated pH levels in the calcifying fluid, even when seawater pH decreases (68, 69). So far, energy turnover rates remain poorly described, which limits our ability to understand and predict the capacity of corals to adjust and respond to environmental change.”

-    Suggestions to improve color schemes of specific figures

**RE**: We improved the color scheme of the figures following recommendations of reviewer #2.

-    Code should be made available (e.g., publication on github or similar repository).

**RE**: The code for the generalized linear models (GAMs) and statistical analyses have now been made available as supplementary information (Script_for_GAMs.txt). Also, the raw metabolic rates are now available as supplementary information and contains the files:

- my_data_resp.csv (all respiration rates)
- fall_NP_algae.csv (all net photosynthesis rates of algae in fall)
- fall_NP_coral.csv (all net photosynthesis rates of corals in fall)
- spring_NP_coral.csv (all net photosynthesis rates of corals in spring)
- summer_NP_coral_v2.csv (all net photosynthesis rates of corals in summer)

The description of the data files is included in “Script_for_GAMs.txt”.

Originally, we planned to publish the results on PANGAEA, but we think it is equally good to be accessible through the supplementary material of this paper. The data sets are comparatively small. Moving forward (with upcoming publications) we will make an effort to use github (or similar).

**Reviewers' comments:**

**Reviewer #1**

This nicely written and concise manuscript mainly aims at unraveling diurnal and seasonal patterns in coral and algae respiration rates. For this, three coral species and three algal species were collected and respiration (and photosynthesis) rates measured at three different timepoints throughout the year. A new way was established to calculate respiration rates, by calculating daily rates instead of assuming night respiration to be constant, and subsequently gross photosynthesis and GP:R ratios. The manuscript finds that traditional rates may underestimate daily R and GP rates, highlighting a more temporal resolution of diurnal changes in oxygen fluxes.

The manuscript is well written and focusing on the main aspects of the work. While the introduction is very concise and to the point, I am missing some references in the text (as can be seen in my detailed comments below). The methods are described well and allow for reproducibility of the setup, though I am missing a bit more detail regarding the incubation time for net photosynthesis rates and the amount of run controls. The results are really nice and supported by well executed supporting figures and tables, including the supplementary material. I am missing statistical tests though to see if the reported differences in oxygen fluxes between the new and traditional method are significant or not, as based on Figure 6 it visually looks like the results may not be significant. The discussion is also written well, missing maybe some references, but I am missing more detail and recommendation in the discussion about Rnew specifically. The study is definitely interesting and has a good basis, but I think by addressing below comments it can be improved. The comments are just ordered by line numbers.

**RE**: Thank you for the overall positive feedback. The shortcomings described in the second paragraph are addressed further below as they are listed at specific sections of the ms.

27-32; 34-38; 40-42; 46-52; 110-112: no references given for those statements

**RE**: We didn’t include references here, as we thought that the statements are so generic and common knowledge. However, we followed your suggestion and added the following references as appropriate:

(1) Kinsey DW. Standards of performance in coral reef primary production and carbon turnover. In: Barnes DJ, editor. Perspectives on Coral Reefs. Townsville, Australia: Australian Institute of Marine Science; 1983. p. 209-20.

(2) Edmunds PJ, Davies PS. An energy budget for *Porites porites* (Scleractinia). Mar Biol. 1986;92(3):339-47.

(3) Muller EB, Kooijman SALM, Edmunds PJ, Doyle FJ, Nisbet RM. Dynamic energy budgets in syntrophic symbiotic relationships between heterotrophic hosts and photoautotrophic symbionts. J Theor Biol. 2009;259(1):44-57.

(4) Leuzinger S, Willis B, Anthony KN. Energy allocation in a reef coral under varying resource availability. Mar Biol. 2012;159(1):177-86.

(7) Muscatine L, editor. The role of symbiotic algae in carbon and energy flux in reef corals: Elsevier, Amsterdam, Vol 25 pp 75-87; 1990.

Also following the suggestion of reviewer #2 we combined paragraph 1 and 2 of the introduction. In doing so we also shortened some statements. The first paragraph of the introduction reads now as followed:

L26-42: “Hermatypic corals and algae are the predominant benthic organisms in coral reefs. Through respiration and photosynthesis, they play a significant role in carbon and energy flow within coral reef ecosystems (1). Like in most organisms, mitochondrial respiration of corals and algae is the metabolic process that transforms stored energy from organic carbon sources like carbohydrates, lipids, and proteins into adenosine triphosphate (ATP), the primary energy carrier within cells. ATP fuels essential cellular activities, including anabolic processes (macromolecule synthesis), gene regulation, transmembrane transport, and cell signaling. Key energy expenditures for corals and algae include cell maintenance, defense mechanisms, biomass growth, calcification (in corals and some algal species), mucus production, and reproduction (2-4). These energy-demanding processes compete with each other, especially during energy scarcity or when environmental stressors increase the demand for mitigation or repair mechanisms (5, 6). The main source of energy in many coral species (2, 7) and the sole source of energy in algae is photosynthesis. As coral reefs face growing exposure to environmental stressors such as pollution, heat waves, and ocean acidification, it becomes crucial to accurately measure energy acquisition and utilization (8). This will improve understanding and predictions of coral resilience as well as potential shifts in the flow of energy and carbon through reef ecosystems.”

**For L46-52 (**now L44-49**)**, we included the following references:

(9) Camp EF, Krause S-L, Santos LM, Naumann MS, Kikuchi RK, Smith DJ, et al. The “Flexi-Chamber”: A novel cost-effective in situ respirometry chamber for coral physiological measurements. PLoS One. 2015;10(10):e0138800.

(10) Sawall Y, Nicosia AM, McLaughlin K, Ito M. Physiological responses and adjustments of corals to strong seasonal temperature variations (20–28° C). J Exp Biol. 2022;225(13):jeb244196.

(11) Sawall Y, Teichberg MC, Seemann J, Litaay M, Jompa J, Richter C. Nutritional status and metabolism of the coral Stylophora subseriata along a eutrophication gradient in Spermonde Archipelago (Indonesia). Coral Reefs. 2011;30:841-53.

**L110-112**: This sentence is our conclusion from the previously discussed papers and approaches in the paragraph above. Therefore, this does not require a reference. We slightly rephrased this sentence to make this more obvious.

L89-90: “To calculate more accurately in-situ energy and carbon budgets for corals and algae and ultimately for reef communities, we need to determine daytime *R* under natural light conditions.”

77-79: did you mean to say “provides R rates closest to the actual GP”? otherwise I am unsure what you want to say about R rates in this kind of experimental setup

**RE:** No, we mean that the GP rates derived from this specific approach are closest to the true GP. We replaced “actual GP” with “true GP” to be more specific. Also, we think there was a confusion. We wrote “provides GP rates closest to actual GP” not “provides R rates closest to the actual GP”

113-116: Does this mean the rocks were also overgrown with other algae and/or organisms beside the dominant algae of interest? Please specify. And if there were also other organisms, besides the algae of interest, on those rocks please elaborate how you accounted for that and how this might influence the measured fluxes.

**RE:** The rocks were comparatively small. Other organisms growing on the rocks contributed <5% and include other fleshy algae, turf algae, crustose coralline red algae, ascidians, and sponges. We added an example picture of each alga species to the supplementary material (Fig. S1) and added a sentence to the revised ms that explains the contribution of the other species.

L112-118:“Algae were collected by removing a total of 27 small “rocks” (calcium carbonate structures) covered by algae, each rock being dominated by one of the three common algal species: *Caulerpa verticillata*, *Ceramium nitens*, and *Laurencia obtusa* (9 rocks per species; Fig. S1). Other species on the rocks contributed less than 5%, including some fleshy algae species, turf algae, crustose coralline red algae, small ascidians, and sponges. These organisms were not removed, as their impact was relatively minor, and attempting to do so would have risked accidentally dislodging the algae of interest.”

Table 1: as the temperature range is quite high, maybe it would be nice to also indicate the mean temperature for the incubation periods (mean temperatures are described in the text line 233-234)

**RE:** We agree and added the mean temperature to the table.

149-151: was there a lack of hobo loggers to put one in each incubation chamber? Or was there another reason (e.g. sufficient replication) so only 7 incubation chambers were continuously monitored?

**RE:** We didn’t have more HOBO loggers, but it also wasn’t necessary as they were only used for spot-checking that the temperature inside the incubation chambers remained constant. We added the following part to the sentence for clarification:

L152-158: “Only 7 HOBO loggers were available, which rotated between the 9 incubation chambers for spot-checking that the temperature remained constant during incubations and to measure light intensity throughout the day.”

161-163: Can the authors please supply the data and analysis to support this claim in the supplementary material? Also, was there a comparison done between potential differences of oxygen flux measurements based on continuous measurements (fall) vs 30s measurements at beginning and end (summer)? Lastly, why was there a change of methods in oxygen flux measurements within the same study (would be interesting to know if comparing methods was a planned part of the study, or if it had different reasons)?

**RE:** Different sensors were used, due to irregular availability of the sensors. While we agree that it would have been better to stick to the same sensors and procedures throughout the experiment, we are also confident that the use of different sensors did not affect our results. We added the data to confirm the comparability of the 2 sensors to the Supplementary Information (Fig. S2 and S3) and indicted the availability of supplementary information in the revised ms.

Also, continued versus 30s measurements in the beginning and the end of an incubation period did not affect the results (Fig. S2). The reason for the different measuring intervals is that in fall we had enough FireSting oxygen sensors to equip each incubation chambers with its own oxygen sensor which allowed continued measurements. In summer, however, we only had one FireString oxygen sensor, which restricted our measurement to the start and the end of each incubation period. We added this information to the revised ms:

L163-165: “Discontinued measurement in summer was due to insufficient amount of oxygen meters available, resulting in having to measure oxygen concentration in one chamber after the other.”

167-169: It is unclear to me how long the specific light and dark incubations were run for. The dark incubations apparently always lasted 30 min (based on line 175), but then the light incubation was how long? And were the light incubations done before or after the respiration measurements?

**RE:** We clarified this in the revised ms. All incubations were run for 30 minutes, but flushing of the chambers occurred only every 1-4 hours. Day-time respiration was measured in between light incubations. This section reads now as followed:

L177-181: “Incubations lasted for 30 minutes. Water was exchanged every 1 to 4 hours: every 2 hours in the morning and late afternoon and every hour between 10:00 and 16:00 hours to avoid extreme oxygen oversaturation. In the night, flushing was conducted every 3 to 4 hours. Day-time respiration was measured in between light incubations by covering the basins with a thick black tarp...”

Following the suggestion of reviewer #2, we also restructured parts of the methods to improve clarity. The above sentence is now under “2.4 Incubation schedule for NP and R measurements.”

181-182: how many controls were run in total? Or if I understood the setup correctly each day 3 corals and 3 algae were tested, leaving space in 3 incubation chambers for controls on each day. Did the authors run 3 controls each day?

**RE:** Sorry, there was a confusion. Per day, we typically incubated 9 coral (or algae) specimen, 3 specimen (=replicates) per coral species (or algal species). We had 3 species of corals and 3 species of algae. The controls were randomly added to the incubation schedule, but not every day. For control incubation, one chamber out of 9 was left coral (/algae free). 4 to 8 controls were run per season. Each control incubation series lasted for an entire day; like the incubations for coral and algae. The change in oxygen of the control chambers was negligible. The paragraph reads now as followed:

L187-192: “Eight to nine specimen of corals or algae were measured per day (2-3 specimen per species), while distributing them randomly between basins and incubation chambers. Each specimen was measured 3 times per season. To account for O2-consuming or producing processes in the water column, 4 to 8 day-long coral- / algal-free control incubations were run per season, confirming negligible rates of water column processes.”

185-190: It would be interesting to know on how many pictures the 3D models relied on (e.g around 100 pictures per colony/rock); also was there any conversion used to account for the structure of the algae growing on the rocks? For example the study of Cardini et al. 2016 used a conversion factor of 3.9 for turf algae surface area measurements ([https://doi.org/10.1007/S10021-016-9966-1](https://urldefense.com/v3/__https:/doi.org/10.1007/S10021-016-9966-1__;!!IKRxdwAv5BmarQ!bg-_SOh2hNXJ0VCExz9PBBt373l6K4jp1GcpperxCJVLU7p2udARMOKWoHeAZxTG-DmHXpMByOV1AiCIDSIILi4$)) and maybe this would be of interest/relevant for the algae species studied here.

**RE:** We noticed a mistake in the description of the surface area calculation or better in the standardization of the metabolic rates. Thank you for making us double check this again. We didn’t standardize the metabolic rates to the 3D, but to the 2D surface area. Originally, we standardized the algal rates to 2D surface area as well as to dry weight biomass, and the coral rates to 3D and 2D surface area. For the 3D surface area of the corals about 20 to 30 pictures were taken (there all have massive growth forms, therefore 20-30 pictures were enough). Finally, we decided to report both algae and coral rates in 2D surface area as (1) it is almost impossible to get realistic 3D surface areas for algae, (2) it is more relevant if those rates were to be used to calculate community rates, where percentage contribution of different benthic species or taxa are derived from common photomosaics or video transects typically capturing 2D surface areas, and (3) because this was the only unifying unit between the algae and corals. Although it can be debated that using 3D surface area and conversion factors for algae would provide a more accurate standardization, this does not ultimately affect the study's outcomes. The study focuses on relative changes in R and P rates over the course of the day and between seasons, making the chosen standardization method suitable to address the objectives.

We fixed the description of the standardization in the ms and we added example pictures with manual outlines that were used for surface area calculations.

L195-201: “Metabolic rates of corals were standardized to the 2-D surface area of coral colonies and to the 2-D surface area of algae assemblages taking pictures from above including a ruler for reference. The pictures were imported to the software ImageJ to determine the surface area (Fig. S1). Although metabolic rates of algae are more commonly standardized to biomass, we decided to use algal 2D surface area to (i) make it more comparable to the coral metabolism rates and, (ii) because community / reef-scale metabolic rate measurements typically rely on measurements standardized to surface area.”

194: missing closing brackets after the reference

**RE:** Fixed.

191-202: I think the calculations would benefit from a visual representation of how they were done, for the readers understanding of the process. Like a schematic representation of the curves, hourly rates, and which rates where summed for R/GPnew vs R/GPtrad calculations for a single incubation chamber over the course of 24h incubations

**RE:** We followed your suggestion and added an illustration that explains the different rates measured and calculated (Fig. 1). We also improved the description of the rate calculations in the revised ms (L202-217).

240-243: as the units between PAR and solar radiation at the weather station are different it is hard for the reader to compare if the values are actually similar or not. Maybe it would be an idea to convert them into the same unit to have a clearer comparison.

**RE:** The 2 types of measurements cannot be compared directly, as they have been measured with different sensors that integrate different wavelengths. The idea here is to give the reader the opportunity to look at seasonal variation in light and to check whether the seasonal variation measured at the mesocosms inside the incubation chambers are comparable to the larger-scale changes in solar radiation to which the organisms are adjusted to. It seems that the seasonal variation in the incubating chambers is a bit stronger than the larger-scale solar radiation measured in air by the Bermuda weather station due differences in partial shading by the experimental infrastructure, which we explained in the ms (L261-268).

302-304: I am not sure if the slope of the declining curve in the afternoon is the main/only characteristic to determine how pronounced the diurnal pattern is. What about the range/amplitude of the curve? If you compare the range of R of Porites between spring (0.72) and fall (0.53), or for Montastrea (spring: 0.81; fall: 0.6) the amplitude is higher in spring, so I would argue that could show a more pronounced diurnal pattern in spring (contrary to what the authors are saying). Though both arguably have a way lower amplitude in spring and fall compared to summer, so maybe this alternative/additional comparison is not as fruitful as I was initially assuming.

**RE:** We have been thinking about the level of detail we want to discuss the shape of the diurnal curve a bit more, and decided that it is better not to go into too much detail, considering the fairly high variability of the raw data. We think the most valuable knowledge gained from the diurnal curves includes (1) the amplitude (difference between min and max R) and (2) the time lag between R and GP. Consequently, we removed the statement “Furthermore, the diurnal pattern (shape of curve) is less pronounced in spring than in fall and summer, which mostly evident in the slope of the declining curve in the afternoon (Fig. 5).”

312-313 and throughout the results: When talking about daily R rates – as Rnew and Rtrad were introduced in the beginning, please always write which R rates are talked about in the results specifically, so the reader knows.

**RE:** We fixed this in the revised ms. We also restructured the results quite a bit to improve clarity following the suggestion of reviewer 2.

318-339: Did the authors test if the reported differences are statistically significant? This analysis would help strengthening their point, rather than just reporting the percent difference. This goes for the comparison specifically of Rnew to Rtrad (and generally all derived values - R, GP, and GP/R – and their new and trad comparison), but could also be done for comparing the difference in GP or GP/R between the different months if this would be of interest. Statistical testing could really boost the paper, and would needed to be included in the Material and Methods and Results.

**RE:** We are not able to conduct statistical tests on the derived values, as they are the product of the GAMs that were fitted across all (!) replicates. As described in the methods section “2.6 curve fitting and error propagation” it was not possible to do GAMs on individual coral or algae specimen as there were not enough data points per specimen/replicate. Consequently, we don’t have derived values, such as GP, GP/R ratios, maximum R, etc. from individuals which precludes statistical analyses.

We tested, however, if the GAM models are significantly different to a straight horizontal line with the p-values being presented in table S1, and now also reported in the revised ms. All diurnal patters of R were significantly different from a straight horizontal line except for *Diploria* in summer. All diurnal patterns of NP were significantly different from a straight horizontal line.

351-354; 420-422; 424-427; 486-489: no references given for those statements

**RE:** We added the following references to the statements.

L351-354 (now L389): We added the same references as in the introduction that relate to this statement.

420-422 (now L463): We added the reference Leach et al. 2018 to this statement.

L424-427 (now L469): We added 2 sentences that show that metabolic rates increase with temperature in corals (Sawall et al. 2022, Edmunds 2005).

486-489 (now L531-542): We rewrote this part of the ms and added a reference following your suggestion further below.

375-377: While the authors are nicely highlighting the previous studies and how their results differ from the increased R rates measured in this study (lines 377-392), I am lacking an explanation or implication of the findings of this study. As the findings are contrary to previous ones how can this difference be explained? Do the new results have specific implications?

**RE:** None of the previous studies investigated respiration under the same conditions we did, meaning under natural light and over entire diel cycles. Therefore, our results are not necessarily contrary to previous findings, but highlight that previous findings don’t tell the full story about coral and algae respiration under natural condition. We clarified this in the revised ms by adding the following sentence:

L428-433: “While most of these studies found a positive relationship between respiration and light, none of them investigated respiration rates over an entire diel cycle under natural light. This likely explains the discrepancy between our and previous results and highlights the importance of considering the natural dynamics of light, if the goal is to gain an understanding of in-situ coral and algae respiration rates and energy consumption.”

408: missing closing brackets after the reference

**RE:** Fixed.

480-489: I am missing more detail and recommendation in the discussion about Rnew and what these potentially higher turnover rates in algae and coral mean. How does this affect current knowledge about energy budgets, etc.?

**RE:** We agree and added some information about the significance of the turnover rates to the revised ms. We modified the last sentence of the paragraph and added more information, as well as a couple of references that help clarifying the connection between energy turn over and resilience. It now reads as followed:

L531-542: “Those turnover rates reflect the scale of metabolic activity, including, for example, energy requirements of transmembrane transporters, movement, and cellular maintenance processes such as protein turnover and repair mechanisms. Quantifying these rates is important because their demand can vary and adjust in response to changing environmental conditions. Species or genotypes with high energy turnover—and/or a greater capacity to regulate their energy turnover in response to changing demands—may be better equipped to cope with environmental stressors such as ocean warming and acidification. For instance, some corals can increase the activity of energy-demanding H⁺-pumps at the site of calcification to maintain elevated pH levels in the calcifying fluid, even when seawater pH decreases (68, 69). So far, energy turnover rates remain poorly described, which limits our ability to understand and predict the capacity of corals to adjust and respond to environmental change.”

514-517: missing more detail about this conclusion in the discussion, as it is never really discussed in light of previous literature.

**RE:** We rephrased the last sentence a bit as followed. It’s essentially summarizing what we concluded 2 paragraphs above, which we improved following your suggestion above (L531-542). The link between energy budgets and resilience should overall be clearer now.

L567-569: “This refined approach holds implications for predicting organism resilience to changing environmental conditions more accurately, as resilience is directly linked to energy availability, energy allocation and turnover.”

**Reviewer #2**

3. Not all data and data analysis steps are made available. In the Supplementary only the summary output of the GAM model and the summary of the metabolic rate can be found. It is not clear whether or not these tables contain all the data that was collected or just an overview. More information in the table description would be useful to clarify this. Furthermore, a Github site containing all of the data as well as the data sheets and codes used for the analysis should be provided.

**RE:** The tables provide statists of all (!) GAMs performed (Fig. S1) and of all mean metabolic rates calculated (Fig. S2). We don’t think it is useful to output more statistical results of the GAMs. All the raw rates (hourly rates) of respiration and net photosynthesis are now available as data files (.csv) and the script used to produce the GAMs and statistics is now available as a text file (Script_for_GAMs.txt) as part of the supplementary information. I’m not yet very familiar with Github, but will make an effort to use this in the future.

1. The beginning of the introduction is not well structured. The first paragraph (line 27-32) of the introduction reads very miss placed. I would move this paragraph down one paragraph and start with a more general introduction first and then go into detail about Respiration and photosynthesis and the current state of knowledge regarding the R and PS dynamics.

**RE**: We followed the suggestion. We incorporated the first paragraph into the second paragraph, and also shortened it a bit for a better integration. (see L26-42)

2. In the material and method part, the sections are not well organized. E.g. Line116-127 rather experimental design than study species. Line 148-155 rather experimental design than metabolic rate measurement, Line 163-165 rather experimental design than metabolic rate measurement. Line 178-179 rather metabolic rate calculation than measurement and also repetition to line 191.

**RE:** We agree that the material and method section was not well organized and restructured it following your suggestion. We also introduced new subheading for improved clarity. The subheadings are now:

- 2.1 Study species,
- 2.2 Coral and algae maintenance,
- 2.3 Experimental setup of incubations,
- 2.4 Incubation schedule for net photosynthesis (NP) and respiration (R) measurements,
- 2.5 Metabolic rate standardization and calculation,
- 2.6 Curve fitting and error propagation.

3. Table1 (line 129-132) and figures: The units should be written in []

**RE:** We added [] in the table, but left the () in the figures, as introducing [] to the code in R caused error messages.

4. The quality of all figures have to be improved. The colors used in the figures are not intuitive.. Furthermore, it is confusing that NP and spring have the same color. The figures could be improved by choosing colors such as: Dark green GP, light green NP, blue R and for the seasons: fall: orange, spring: yellow, summer: red. The figure descriptions have to be extended to a degree that the figure and the description can stand alone to understand the plot. Therefore, I would recommend to add more information and also the main result that is shown in the plot as the title of the figure.

**RE:** We improved the figures by using more intuitive colors and also did some other small adjustments to improve clarity. We added more information to the figure captions, but did not add titles to the figures, as we believe that the improved figure captions and the overall better structure of the results including subheadings will be sufficient.

5. The result section could also be structured better, by dividing the metabolic rates section into diurnal and season or by respiration and photosynthesis results. As title for each section the main finding should be used to clearly stating the result.

**RE:** We agree. We added subheadings, restructured part of the results and also rewrote sections to improve clarity. The subheadings are as followed:

- 3.1 Environmental conditions,
- 3.2 Diurnal pattern of respiration (R) in algae (fall) and corals (fall, spring, summer)
- 3.3 Relating diurnal pattern of respiration (R) to photosynthesis (NP, GP)
- 3.4 Daily rates of respiration (Rnew) and gross photosynthesis (GPnew) in algae (fall) and corals (fall, spring, summer)
- 3.5 Comparing “new” and “traditional” daily respiration rates (Rnew versus Rtrad) and implications for daily gross photosynthesis rates (GPnew versus GPnew)

6. Was the symbiont density also investigated? In line 267 you mention that: "Algal assemblages had a higher R as corals during the fall .." Is this only due to higher metabolic efficiency of the algae compared to the coral or could it also be due to differences in algae density compared to the coral? the difference between algae and coral would also be interesting to discuss in the discussion section.

**RE:** No, we did not investigate symbiont density. In this sentence we referred to the investigated macroalgal species (Caulerpa, Laurencia and Ceramium). We changed “algal assemblages” to “algae species” to avoid confusion. The difference between algae and coral rates is likely driven by standardization. Both rates are standardized to 2D surface area. I assume that there is more biomass of algae then of zooxanthellae in corals per surface area. See our addition to the supplementary information about the surface area calculation of algae (Fig. S1). I think, it may have been interesting to standardize the rates to unit of chl-a to evaluate photosynthetic efficiency (which we are not able to do anymore).

7. The explanation of higher R in line 377 to 392 is very extensively described by stating the exact results of other studies and there measured values. This section could be shortened by focusing on one example specifically and only briefly mentioning the others or by focusing on the main differences and discussing it in comparison to this study.

**RE:** The reason why we included several examples is that we wanted to show the diversity of approaches used - mostly different light regimes - and also the fact that different organisms have been investigated (corals and algae). We only found one macroalgae example therefore we also included phytoplankton examples. We prefer to keep all examples, as they provide a better justification why our study is different. Following a suggestion of reviewer #1, we added some information to the revised ms that clarifies how and why our results differ from previous studies. (see L428-433)
