# Supplementary material for: Diurnal pattern of respiration in corals and algae and its implications for gross primary production quantification
Source: PLoS One. 2025 Jun 27;20(6):e0326146. doi: 10.1371/journal.pone.0326146 (PMC12204508; doi:10.1371/journal.pone.0326146)
Supplement: Attachment — Submitted filename: Rebuttal Letter PONE_2.docx [file pone.0326146.s006.docx]

May 2025

**Rebuttal Letter 2, PONE-D-24-55887**

**Reviewer #1:** The authors have answered all my questions and comments fully in a nice, understanding, and concise way. I fully support this manuscript to be published.

I only found 3 minor formatting "issues" that should be corrected in editing:
Line 202: "were" different font

**RE**: Fixed. Can’t believe that you even found this :)

Fig. 2 description: different font sizes

**RE**: Fixed.

Fig. 3, 4, and 5 description: "confidence interval" in different font size

**RE**: Fixed.

**Reviewer #2**: 1) The authors have adequately addressed the comments raised by reviewer 1 and 2 and incorporated the highlighted the raised comments well into the revised manuscript.
2) The manuscript is now more technically sound and the data is now better presented and explained.
3) The statistical analysis has been performed appropriately and the data description and presentation is now improved by the additional explanations and changes in the manuscript.
4) The underlying data is now better accessible and fully available.
5) yes, but the manuscript should be checked again for grammatical error since some minor errors can be found e.g. line 301.

**RE**: We did a thorough read through the ms again and fixed a few minor errors.

6) additional comments
- In the description of fig.1 the font and size of the text changes.

**RE**: Fixed.

- In figure 5 the GP and NP of the corals plots is not correctly colored. Adjust color legend or adjust figure.

**RE**: We removed the color legend and described the plots and colors in the figure caption.

- The quality of the figures is still quite bad. Please improve for final manuscript.

**RE**: We did some further improvements to the quality of the figures.

- In the new figure 1 the unit of the y-axis is not provided, please make sure that units are presented in squared brackets [].

**RE**: We added the units in square brackets. We also modified the other figures in terms of units in squared brackets.
